# Access to primary care psychological therapy services for people living with dementia

**DOI:** 10.1002/alz.086920

**Published:** 2025-01-09

**Authors:** Celine El Baou, Rob Saunders, Joshua Buckman, Marcus Richards, Georgia Bell, Caroline Fearn, Claudia Cooper, Stephen Pilling, Sebastian J Crutch, Nikki Zimmermann, Emilie V Brotherhood, Amber John, Joshua Stott

**Affiliations:** ^1^ University College London, London United Kingdom; ^2^ Camden and Islington NHS Foundation Trust, London United Kingdom; ^3^ MRC Unit for Lifelong Health & Ageing at UCL, London United Kingdom; ^4^ Queen Mary University of London, London United Kingdom; ^5^ University College London, London, London United Kingdom; ^6^ Dementia Research Centre, UCL Queen Square Institute of Neurology, London United Kingdom; ^7^ Dementia Research Centre, UCL Queen Square Institute of Neurology, University College London, London United Kingdom; ^8^ Department of Clinical, Educational, and Health Psychology, Division of Psychology and Language Sciences, University College London, London United Kingdom

## Abstract

**Background:**

Psychological therapies are recommended for people living with dementia who experience depression or anxiety. However, people living with dementia often experience specific barriers to accessing services providing such interventions, notably due to the stigma associated with dementia. This study sought to understand pathways to entering and receiving treatment after referral to primary care psychological therapy services for people living with, versus without dementia.

**Method:**

Linked national data from England from over 4 million individuals referred to NHS Talking Therapies for anxiety and depression (NHS TTad) services were used to identify 6623 people living with dementia referred to these services between 2012 19. We compared rates of access and entry into treatment between people living with dementia and a propensity score matched cohort of people without a diagnosis of dementia, adjusting for a range of socio‐demographic (age, gender, ethnicity) and therapy factors (e.g waiting times).

**Result:**

We found that once people living with dementia were referred to NHS TTad, they were less likely to access services (ie actually receive an assessment) [AdjOR = 0.61 (0.56; 0.67) p<.0001]. Once assessed, they were also less likely to receive treatment [AdjOR = 0.68 (0.62;0.75, p<.0001] than people with no diagnosis of dementia. Services were twice as likely to be deemed “not suitable” (based on discharge codes recorded by clinicians) for people living with dementia [AdjRRR = 1.97 (1.68; 2.30) p<.0001]. were similar between typical (e.g., memory‐led Alzheimer’s disease) dementias, and atypical dementias (e.g. frontotemporal dementia).

**Conclusion:**

This study highlights inequalities in access to psychological therapies for people living with dementia. There is a need to make services more accessible to improve the rate of treatment initiation for people living with dementia that are referred and assessed for psychological therapies.

